# Comparison of Surgical Treatment Outcomes in Patients with Symptomatic Severe Aortic Valve Stenosis Using the Perceval Sutureless Bioprosthesis Versus a Conventional Biological Valve

**DOI:** 10.3390/jcdd12080308

**Published:** 2025-08-13

**Authors:** Dejan M. Lazović, Milica Karadžić, Filip Vučićević, Gorica Marić, Miloš Grujić, Ivana Đurošev, Mladen J. Kočica, Svetozar Putnik, Dragan Cvetković

**Affiliations:** 1 Clinic for Cardiac Surgery, University Clinical Center of Serbia, 11000 Belgrade, Serbia; milica.karadzic@gmail.com (M.K.); dr.milosgrujic@gmail.com (M.G.); ivana.djurosev@gmail.com (I.Đ.); svetozar.073@yahoo.com (S.P.); d.cvetkovic@sezampro.rs (D.C.); 2 Faculty of Medicine, University of Belgrade, 11000 Belgrade, Serbia; goricamaric87@gmail.com; 3 Center for Anesthesiology, Reanimatology and Intensive Care Medicine, University Clinical Center of Serbia, 11000 Belgrade, Serbia; 4 Institute of Epidemiology, Faculty of Medicine, University of Belgrade, 11000 Belgrade, Serbia

**Keywords:** aortic valve stenosis, surgery, aortic valve replacement, heart valve prosthesis, bioprosthesis, sutureless bioprosthesis, Perceval valve, conventional stented valve, survival rate, retrospective comparative cohort study

## Abstract

**Objectives:** This single-center retrospective comparative cohort study aimed to compare the outcomes of aortic valve replacement using a Perceval sutureless bioprosthesis versus a conventional stented bioprosthesis in patients with symptomatic severe aortic valve stenosis. **Methods:** A total of 233 consecutive elective patients undergoing aortic valve replacement (AVR) at the University Clinical Center of Serbia (July 2017–March 2021) were analyzed: 74 received a Perceval sutureless valve, and 159 received a conventional stented valve. **Results:** The baseline characteristics were similar between the groups, with most patients being male (54.1% vs. 56.6%), with a mean age of 72.6 years. Combined aortic valve replacement and coronary artery bypass grafting were performed in 19.3% of the patients. Mean aortic cross-clamp (ACC) time was significantly shorter in the Perceval group for combined procedures (104.5 ± 29.6 min, *p* < 0.05) but similar in isolated AVR, likely reflecting the early institutional learning curve. Thirty-day mortality was comparable (5.9% vs. 6.3%). Importantly, at 36 months, survival was higher in the Perceval group (88.3% vs. 76.8%, *p* = 0.048). Longer echocardiographic follow-up (up to 58 months) was available for the Perceval group. **Conclusions:** Perceval sutureless bioprostheses are a safe and effective option for elderly high-risk patients. The extended echocardiographic follow-up represents a novel contribution to the literature, although further data on long-term durability are needed.

## 1. Introduction

In developed countries, aortic valve stenosis (AS) is the most prevalent valvular heart disease [1]. Patients with severe AS and decompensated heart failure may experience transient improvement with vasodilator therapy as a bridge to aortic valve replacement (AVR) [2,3]; however, there is no effective long-term medical therapy for AS.

Surgical AVR is the gold-standard treatment for severe symptomatic AS [4]. In asymptomatic patients with severe aortic stenosis (AVA ≤ 1.0 cm^2^, peak velocity ≥ 4.0 m/s, or mean gradient ≥ 40 mmHg), intervention is recommended when high-risk features are present and surgical risk is low. These include left ventricular ejection fraction LVEF < 55% without alternative cause, very severe AS (peak velocity > 5.0 m/s, rapid progression, or elevated brain natriuretic peptide (BNP), abnormal exercise testing, systolic pulmonary artery pressure (sPAP > 60 mmHg), or the need for concomitant cardiac surgery [5].

AVR has been performed since the 1950s [6]. Over time, increasing patient age and comorbidities have prompted refinements in surgical techniques and valve design to reduce procedure-related complications. One key goal is to minimize the operative time, as prolonged cardiopulmonary bypass (CPB) and aortic cross-clamp (ACC) durations are associated with higher morbidity and mortality [7,8]. However, early experiences with sutureless valves may not consistently achieve shorter operative times due to the learning curve associated with their adoption.

As the population ages, the prevalence of degenerative AS that requires surgery continues to increase. However, advanced age and cumulative comorbidities place approximately 25% of patients in the high-risk category for conventional AVR with sutured prostheses (mechanical or stented biological valves). This is especially true in patients with heavily calcified or small aortic roots, impaired left ventricular function, or those requiring concomitant procedures [5,9].

Sutureless aortic bioprostheses have been developed to simplify technically difficult and time-consuming AVR procedures in high-risk patients to reduce operative times, morbidity, and mortality. These valves allow rapid deployment under direct vision and provide a larger effective orifice area (EOA) for a given annulus size, combining the advantages of conventional surgical AVR (complete excision of the diseased valve under direct visualization) with those of transcatheter AVR (elimination of sutures and shorter implantation time) [10,11,12]. Sutureless AVR (SU-AVR) has demonstrated favorable early outcomes in high-risk and combined-procedure patients [13]. The successful implementation of the Perceval sutureless valve is closely tied to the operator’s experience and the institutional learning curve [14].

The Perceval sutureless aortic valve (Corcym, Italy) is a collapsible, self-expanding bioprosthesis implanted in more than 22,000 patients worldwide over the last decade [15]. In appropriately selected patients with severe AS (including those with combined stenosis and regurgitation or a failing aortic prosthesis), Perceval has proven to be a safe and effective alternative to standard AVR via either full sternotomy or minimally invasive approaches in appropriately selected patients with severe AS, including those with combined stenosis and regurgitation or a failing aortic prosthesis. The valve is constructed from bovine pericardium, with tissue treated with glutaraldehyde and homocysteic acid to mitigate calcification and fixed within an expandable nitinol stent frame that secures the bioprosthesis at the native annulus. Intra- and supra-annular sealing collars help minimize paravalvular leaks. Perceval is available in four sizes (small, medium, large, and extra-large) to accommodate annular diameters ranging from 19 mm to 27 mm [16,17,18].

## 2. Materials and Methods

This is a retrospective comparative cohort study based on a prospectively maintained single-center registry, with standardized data collection and follow-up protocols. Although the analysis was conducted retrospectively, all clinical, operative, and follow-up data were collected in real-time as part of routine institutional practice using standardized protocols. This approach aligns with the recommendations for observational research outlined in the STROBE (Strengthening the Reporting of Observational Studies in Epidemiology) statement [19].

Between July 2017 and March 2021, 512 patients who underwent surgical AVR using bioprosthetic valves at our institution were prospectively enrolled. After applying the study inclusion and exclusion criteria, 233 consecutive patients were identified for analysis, including 74 who received a Perceval sutureless valve (sutureless group) and 159 who received a conventional stented biological valve (stented group). The study did not include randomization or propensity matching. The choice to implant a sutureless valve was guided by anatomic feasibility and prosthesis availability rather than random allocation. Patients were selected for sutureless aortic valve implantation based on both institutional protocol and anatomical suitability for the Perceval prosthesis. Eligibility criteria included an aortic annulus diameter between 19 and 27 mm (as measured by intraoperative sizing or preoperative imaging), tricuspid valve morphology in most cases, absence of extensive annular or root calcifications, a non-dilated ascending aorta (<40 mm), and no significant sinotubular junction (STJ) discrepancy (STJ/annulus ratio > 1.3–1.5). Final decisions were made intraoperatively based on surgical judgment and the availability of the sutureless prosthesis. Patients who did not meet these anatomical prerequisites or required complex aortic root procedures were treated with conventional sutured bioprostheses. This study was approved by the Ethics Committees of the UC Clinical Centre of Serbia (protocol code: 111/8, date of approval: 6 April 2021) and of the Medical Faculty, University of Belgrade (protocol code: 17/I-19, date of approval: 12 January 12 2023). Written informed consent was obtained from all the patients.

**Inclusion criteria:**

- Indication for elective AVR with a bioprosthesis (via full sternotomy, mini-sternotomy, or right anterior mini-thoracotomy);
- Age > 65 years;
- Severe AS with New York Heart Association (NYHA) class ≥ II symptoms;
- Critical aortic stenosis on preoperative echocardiography: aortic valve area ≤ 1.0 cm^2^ (or indexed area < 0.6 cm^2^/m^2^), mean gradient > 40 mmHg, peak velocity > 4 m/s, or Doppler velocity index < 0.25;
- Sinotubular junction-to-annulus diameter ratio ≤ 1.3;
- Aortic root dimensions suitable for a Perceval valve (annulus 19–27 mm);
- Signed informed consent.

**Exclusion criteria:**

- Urgent or emergency cases;
- Concomitant procedures other than CABG (e.g., other valve or ascending aorta surgeries);
- Presence of an ascending aortic aneurysm or dissection;
- Congenital unicuspid or bicuspid aortic valve (Sievers type 0);
- Sinotubular junction-to-annulus diameter ratio > 1.3;
- Aortic annulus size < 19 mm or >27 mm;
- History of ST-elevation myocardial infarction (STEMI), Non-ST-elevation myocardial infarction (NSTEMI) or stroke within the last 30 days;
- Active endocarditis, myocarditis, or sepsis;
- Cardiogenic shock necessitating mechanical support;
- Known allergies to nickel or nickel–titanium alloys;
- Inability to provide informed consent.

The baseline preoperative characteristics (Table 1) were recorded, including the patients’ clinical profiles and standard laboratory values. In the conventional stented group, the following biological valves were used: Trifecta (Abbott—formerly St. Jude Medical, St. Paul, MN, USA), Epic Max (Abbott, St. Paul, MN, USA), Crown PRT (LivaNova—formerly Sorin Group, Milan, Italy), Hancock II (Medtronic, Minneapolis, MN, USA). The tissue type (bovine/porcine) and manufacturer-reported EOA for each size were documented (Supplementary Materials: Table S1). All patients underwent preoperative transthoracic echocardiography (TTE) and subsequently intraoperative transesophageal echocardiography (TEE). In patients who underwent minimally invasive approaches (39 patients for upper mini-sternotomy and six for right anterior mini-thoracotomy), a preoperative multi-detector computed tomography (MDCT) scan was performed to assess anatomical suitability (aortic root position, distance from the sternum, and aortic angulation).

### 2.1. Operative Technique

**Figure 1:**
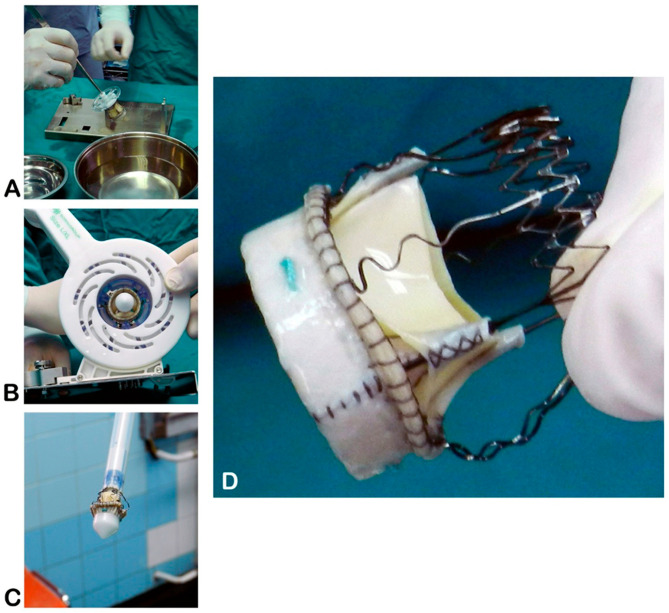
Sterile preparation of the Perceval sutureless valve on the side table. (**A**)—Valve unpacking; (**B**)—collapsing the prosthesis onto the holder; (**C**)—collapsed prosthesis mounted on the holder, ready for implantation; (**D**)—key components of the Perceval device (source: Clinic for Cardiac Surgery, University Clinical Center of Serbia, 11000 Belgrade, Serbia).

All surgeries were performed under general anesthesia with standard monitoring. In 188 cases (80.4%), full median sternotomy was performed, whereas the remaining patients underwent upper mini-sternotomy or right anterior mini-thoracotomy, as determined by MDCT screening. Standard cardio-pulmonary bypass (CPB) was established with central aortic and right atrial (or femoral) cannulation, and myocardial arrest was achieved using cold crystalloid St. Thomas Cardioplegia. A transverse aortotomy was performed approximately 5 mm above the sinotubular junction (near the Rindfleisch’s ridge) to excise the native aortic valve and thoroughly debride the annulus. Three guiding 4-0 polypropylene sutures were placed at the nadir of the aortic sinuses (120° apart) to aid prosthesis alignment. The appropriate Perceval valve size was selected using the manufacturer’s sizers, and the prosthesis was collapsed and mounted on a delivery holder (Figure 1).

**Figure 2:**
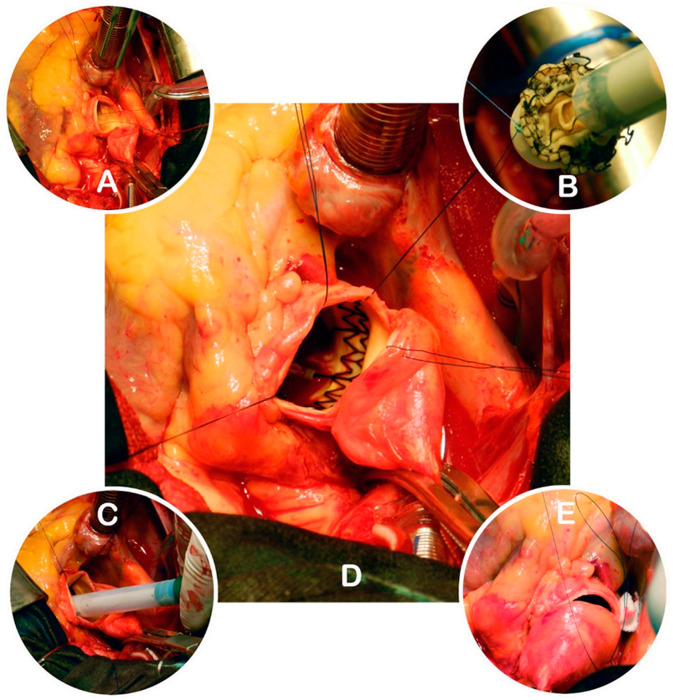
Key steps of Perceval valve implantation. (**A**)—Aortotomy, native valve excision, and placement of guiding sutures; (**B**)—connecting the guiding sutures to the collapsed prosthesis; (**C**)—positioning the prosthesis in the annulus; (**D**)—expanded Perceval valve in situ; (**E**)—closure of the aortotomy (source: Clinic for Cardiac Surgery, University Clinical Center of Serbia).

A sutureless valve was implanted using three guiding sutures that were passed through the annular eyelets of the prosthesis to position it in the annulus. The inflow and outflow frames of the stent were released, allowing the valve to expand and sit in place. Proper positioning and seating were visually confirmed, and the valves were secured. Post-implantation modeling was performed by inflating a balloon inside the valve to four atmospheres for two cycles of 30 s each, with warm saline infused into the aortic root during inflation. The guiding sutures were removed, and the aortotomy was closed in a standard fashion (Figure 2). After weaning from CPB, TEE was repeated to verify the correct prosthesis position and check for any paravalvular leak [18].

**Figure 3:**
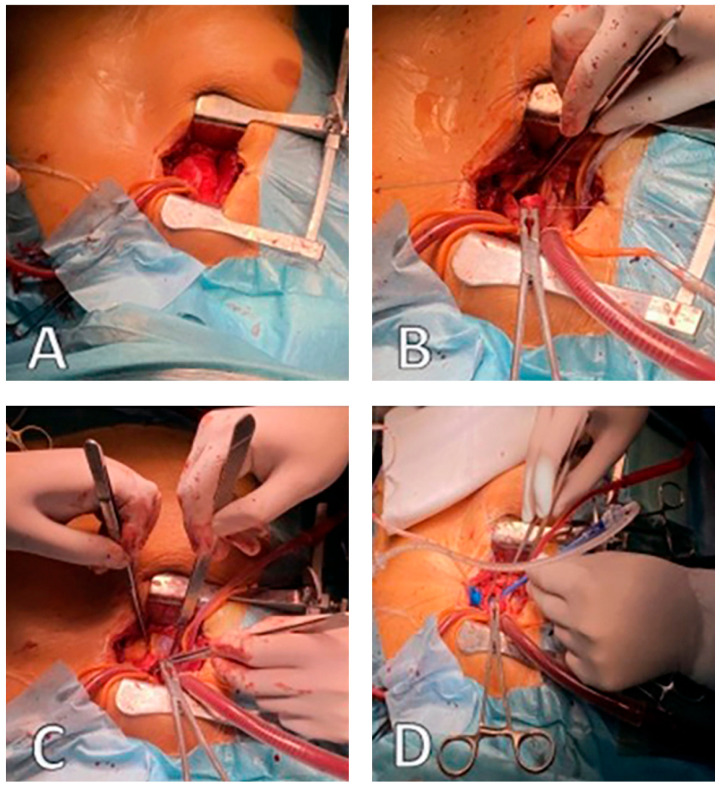
Operative field view during MI-AVR: (**A**)—central cannulation ascending aorta, (**B**)—superior vena cava cannulation, (**C**)—conventional transverse aortotomy, (**D**)—balloon dilation Perceval valve (source: Clinic for Cardiac Surgery, University Clinical Center of Serbia).

Minimally invasive aortic valve replacement (MI-AVR) with a Perceval prosthesis (Figure 3), in selected cases, is usually performed via an upper mini-sternotomy or right anterior thoracotomy (5–7 cm incision) [14,17,18].

Postoperative management was performed according to standard protocols. Patients were monitored in the intensive care unit and then in the surgical ward. Clinical and echocardiographic follow-up examinations were scheduled at hospital discharge and 1, 3, 12, 24, 36, 48, and 58 months after surgery. At each follow-up visit, TTE was performed to evaluate prosthetic valve function (peak and mean transvalvular gradients) and to detect any complications, such as paravalvular leak, valve migration, structural degeneration, or thrombosis. All patients in the Perceval group completed follow-up through 58 months. However, only 36 months’ data were available for the stented group due to administrative and logistical factors. No patients were lost to follow-up in the Perceval cohort. The mean follow-up duration was 42 months (range: 36–58 months).

### 2.2. Statistical Analysis

Data were analyzed using SPSS Statistics v22.0 (IBM Corp.). Continuous variables are presented as mean standard deviation, and categorical variables as absolute counts and percentages. Group comparisons were conducted using Student’s *t*-test for normally distributed continuous variables and Mann–Whitney U test or Kruskal–Wallis test for non-parametric variables, as appropriate. Survival was analyzed using the Kaplan–Meier method and compared between the groups using the log-rank test. A Cox proportional hazards regression model was used to evaluate the effects of the covariates on survival. Multivariate analysis was performed to identify independent predictors of survival in both groups.

## 3. Results

The two groups were well matched in baseline characteristics (Table 1). Groups were similar in age (72.6 ± 7.2 vs. 72.7 ± 7.1 years, *p* NS), sex distribution (54.1% vs. 56.6% male, *p* NS), and body mass index. The mean preoperative transvalvular gradient was 52 ± 17 mmHg in both groups. The Perceval group preoperatively had a smaller mean AVA (0.61 ± 0.15 cm^2^) than the stented valve group (0.90 ± 0.24 cm^2^, *p* < 0.05). The lower AVA in the Perceval group likely reflects more severe AS at presentation and higher afterload. LVEF and gradients were comparable, suggesting that the difference was not due to low output states. The most common Perceval sizes were medium (21–23 mm, 27.0%) and large (23–25 mm, 40.5%), with a mean annular diameter of 22.1 ± 1.7 mm. In contrast, the stented group included a broader size distribution, with a larger proportion of 21 mm (39.6%) and 23 mm (22.0%) valves, and a mean annular diameter of 23.4 ± 1.6 mm (Supplementary Materials—Table S1). There was no significant difference of annular diameters measured by preoperative CT between groups. The prevalence of most comorbid conditions was comparable between the groups, except for hypertension and dyslipidemia, which were more frequent in the Perceval group (94% vs. 76% for hypertension and 66% vs. 40% for dyslipidemia; both *p* < 0.05).

The intraoperative and early postoperative outcomes are shown in Table 2. For patients undergoing combined AVR + CABG, the CPB time was longer in the sutureless group (120.3 ± 38.2 min) compared to the stented group (101.4 ± 36.5 min, *p* < 0.05). However, for isolated AVR procedures, the CPB times were similar between the two groups. The ACC time for the combined procedures was significantly shorter in the Perceval group (92.1 ± 29.3 min vs. 104.5 ± 29.6 min, *p* < 0.05). In isolated AVR procedures, ACC times were comparable between groups. The surgical approach (full sternotomy vs. mini-sternotomy) did not significantly affect the CPB or ACC times in either group. No annular enlargement was performed in this series. Echocardiographic and CT imaging to determine annular dimensions, predicted indexed EOA (iEOA) calculation, and appropriate valve sizing likely eliminated the need. Aortic valve calcium scores were not routinely quantified. No significant intergroup differences were observed in postoperative complications (Table 2). Notably, the rates of stroke (0% in both groups) and permanent pacemaker (PM) implantation (5.4% vs. 3.1%, *p* NS) were low and similar. Early postoperative blood loss, intensive care unit (ICU) stay, and total hospital length of stay were also comparable. Thirty-day (hospital) mortality rates were 5.9% (4 patients) in the sutureless group and 6.3% (10 patients) in the stented group (*p* NS).

Follow-up data were complete for all Perceval group patients through 58 months, with no losses. The stented bioprosthesis group had data up to 36 months due to administrative constraints, not patient attrition. Thus, no follow-up data were missing in either group, and no imputation techniques were necessary. The Kaplan–Meier estimates accurately reflect the longitudinal data for each group. Clinical and echocardiographic follow-up data are presented in Table 3. Over the 36-month follow-up period, there were no differences between the groups in NYHA functional class or prosthetic valve hemodynamics. Most surviving patients in both groups had NYHA class I or II, with no significant intergroup differences (*p* NS). Echocardiography showed low transvalvular gradients in both groups (mean gradient: 9 ± 2 mmHg in the sutureless group vs. 10 ± 2.2 mmHg in the stented group, *p* NS). Based on echocardiographic follow-up (iEOA), we found no cases of moderate or severe patient–prosthesis mismatch (PPM) in either group. The incidence of late complications was generally low. No stroke occurred in either group during the follow-up. There were no cases of endocarditis in the Perceval group and three cases (1.9%) in the stented valve group (*p* NS). The only significant difference was the higher rate of late thrombocytopenia in the Perceval group (20.3% vs. 10.7%, *p* < 0.05), although this did not translate to adverse clinical outcomes (5.9% vs. 6.3%).

**Figure 4:**
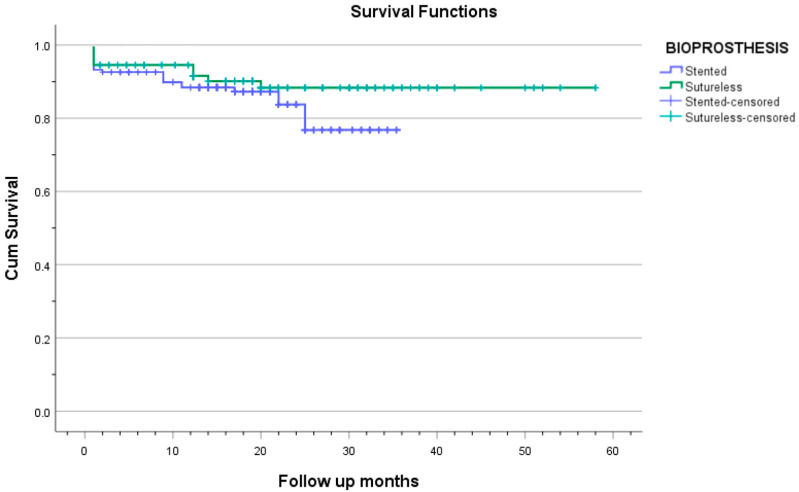
The Kaplan–Meier curve shows the survival of patients in both groups.

Survival comparison between the sutureless and stented groups is reported only up to 36 months, for which complete follow-up data are available for both groups. Kaplan–Meier survival analysis revealed a trend toward better mid-term survival in the sutureless group. At 36 months postoperatively, survival was 88.3% in the Perceval group and 76.8% in the stented valve group (log-rank *p* = 0.048; Table 4 and Figure 4). The survival rate in the Perceval group remained 88.3% up to 58 months postoperatively, whereas no follow-up data were available for the stented group beyond 36 months.

The analysis of mortality causes over time shows distinct trends explaining the survival curve differences between groups. In the first 6 months, deaths were mainly due to cardiac complications like low cardiac output syndrome, arrhythmias, or perioperative myocardial infarction. Afterward, from 6 to 24 months, deaths in the stented valve group largely stemmed from non-cardiac issues such as malignancies, frailty, and sepsis, indicating comorbidities rather than prosthesis problems. Conversely, the Perceval group had fewer deaths during this period, likely due to the less-invasive sutureless valve technique and shorter procedural times in survivors, enhancing recovery for high-risk patients.

## 4. Discussion

Sutureless aortic valve replacement (SU-AVR) offers potential advantages for high-risk surgical patients, especially those with expected prolonged operative times. The Perceval valve simplifies implantation and may reduce cross-clamp time in complex procedures like AVR with CABG. Shrestha et al. emphasized that the need for coronary revascularization in AS patients has risen from 5% to 25% over the last 20 years, highlighting the importance of efficient AVR techniques, finding SU-AVR safe and effective in combined cardiac procedures [20].

The Perceval sutureless valve can simplify procedures and reduce operative times, but these benefits depend on the surgeon’s experience and familiarity with the device. The learning curve is crucial for realizing Perceval’s potential, particularly in achieving shorter ACC and CPB times. Initially, a lack of familiarity with valve sizing, deployment techniques, and device nuances may prolong operations and increase variability. Studies show that as teams gain experience, ACC and CPB durations consistently decrease, improving efficiency, especially in minimally invasive and combined procedures. Recognizing and accounting for the learning curve is essential when interpreting early outcome data and benchmarking performance across centers [14,21,22].

By eliminating the sewing ring and allowing supra-annular positioning, the Perceval sutureless valve offers a larger EOA for a given annulus size than conventional sutured bioprostheses (Supplementary Materials: Table S1). This advantage is especially beneficial for patients with small aortic roots, who face an increased risk of PPM [23,24]. As a result, despite smaller annuli in the Perceval group, postoperative gradients were low and comparable with the stented valve group. No patients required aortic annular enlargement procedures, likely because valve sizing and preoperative imaging ensured annular compatibility. Given the small sample and careful patient selection, this finding may not reflect the broader AVR population, where annular enlargement is more frequently needed to prevent PPM. In patients with borderline annulus dimensions, preemptive annular enlargement should be considered. The posterior root enlargement technique described by Yang et al. offers a reproducible approach to avoid mismatch [25,26].

SU-AVR is also beneficial for minimally invasive surgeries, where limited space complicates suturing. A sutureless prosthesis can be deployed more easily through a small incision, potentially expanding the applicability of less-invasive approaches [14,27,28,29,30]. Using the Perceval valve in minimally invasive cases (upper mini-sternotomy or anterior thoracotomy) was feasible without prolonging our cohort’s CPB or ACC times.

Another important consideration is the operative time during combined and complex procedures. Prolonged ACC and CPB times are known to increase morbidity, particularly in elderly patients and those with multiple comorbidities (reflected by high EuroSCORE-II and STS risk profiles) [31]. Utilizing a sutureless valve in such combined and complex procedures could reduce ACC and CPB times [32,33]. Operative times (ACC and CPB) in isolated AVR were comparable between our sutureless and stented groups. Yet, in the combined AVR + CABG, the sutureless group had significantly shorter ACC time and significantly longer CPB time. The former may reflect an early institutional learning curve, and the latter likely reflects greater procedural complexity in these specific cases, including multi-vessel grafting or anatomical challenges, as well as setup and weaning phases that extend beyond the clamping phase. In the largest reported series of combined procedures to date, Shrestha et al. documented mean ACC and CPB times of 51 ± 23 min and 79 ± 32, respectively, using the Perceval valve [20], supporting that sutureless AVR is closely tied to the operator’s experience and the institutional learning curve.

A potential drawback of Perceval’s prosthesis is its long-term durability. Englberger et al. reported the 5-year outcomes of an earlier-generation sutureless valve, suggesting that these prostheses may not be suitable for all patients who are candidates for bioprosthetic AVR [34]. Further research and more extended follow-up periods are needed to determine the durability and performance of Perceval over time.

Limited data on using sutureless valves in patients with bicuspid aortic valves are available. Initially, bicuspid anatomy (especially Sievers type 0) was considered a contraindication for SU-AVR because of its elliptical annular shape. However, subsequent reports have described successful percutaneous implantations in patients with bicuspid AS without increased complication rates. Nguyen et al. reported 25 bicuspid patients with no intra- and postoperative paravalvular leakage, valve migration, or embolization. Patients with Sievers type 0 bicuspid aortic valves were excluded. Selected Sievers type 1 patients were considered eligible if the raphe was non-calcified and annular geometry allowed adequate Perceval seating. Only two patients (2.7%) had a bicuspid aortic valve (Sievers type 1) in our cohort, and both received Perceval with no device-related issues [24,35].

Conduction disturbances remain relevant following Perceval valve implantation, primarily due to the radial stress exerted by the self-expanding nitinol stent on the conduction system. Early single-center series reported complete atrioventricular block requiring permanent PM in approximately 9–10% of patients, with pre-existing right bundle branch block (RBBB) and valve oversizing strongly predictive of pacemaker need [36]. A multicenter pooled meta-analysis including 9492 patients found an overall permanent PM implantation rate of 7% (95% CI 6–9%), with a trend toward decreased rates over time as experience increased [37]. In newer cohorts, technical refinements, especially avoiding oversizing and precise annular placement, have reduced PM rates from around 11% to 6% [10]. Risk factors such as older age, pre-existing conduction disturbances, thickened septum, bicuspid aortic valve, and concomitant mitral or tricuspid valve procedures were identified in some studies as predictors of PM requirement after AVR [38,39,40,41]. The permanent PM implantation rate in our study was 5.4%, which is within the range reported in the literature for sutureless valves.

In a comprehensive mid-term evaluation involving 468 patients who received Perceval valves, the observed 30-day mortality rate was 3.2%, increasing to 8.8% at one year. Notably, none of the reoperations were attributed to paravalvular leak (PVL) or structural valve deterioration [40]. A systematic review encompassing 2505 patients reported 30-day mortality rates ranging from 0% to 4.9%, with moderate-to-severe PVL observed in up to 8.6% of cases and reoperation rates reaching 4.8% [42]. A large meta-analysis of 3196 patients over five years demonstrated a lower pooled 30-day mortality of 2.5%, with severe PVL and structural valve deterioration requiring reoperation being rare at 1.6% and 1.5%, respectively [43]. Additionally, a Japanese post-marketing surveillance study of 204 high-risk patients reported an extremely low 30-day mortality of 0.5%, rising to 4.4% at one year, with no cases of moderate or severe PVL necessitating reoperation [44]. These findings collectively indicate that early mortality following Perceval valve implantation ranges from 1% to 4%, tends to decrease over time, with one-year mortality generally below 9%, and that severe PVL requiring reoperation remains infrequent, occurring in less than 2% of large patient cohorts. Our study reported a hospital and 30-day mortality rate of 5.9%, and a 1-year mortality rate of 8.4%. The incidence of significant PVL requiring reoperation in our series was 4.0%, which is slightly higher than that reported in most published reports on sutureless valves [16,40,42,43,44].

This study has several limitations due to its retrospective, single-center design and the absence of randomization or propensity score matching, which constrain the ability to draw broad generalizations or establish causality. While the comparative analysis between sutureless and sutured groups offers valuable information, the use of unmatched cohorts introduces potential for selection bias concerning baseline risk factors and surgical indications. No preoperative variable was significantly associated with mortality in univariate analysis, and the low number of events would not permit statistically valid multivariate modeling. Although baseline demographics, EuroSCORE-II, and STS scores (Table 1) allowed reasonable comparability, residual confounding cannot be excluded and may partially contribute to observed survival differences. Larger prospective or randomized studies will be required to identify independent predictors of long-term survival. Techniques such as propensity scoring could enhance comparability, but the relatively small sample size, particularly in the sutureless valve group, limits statistical power and increases the risk of overfitting. A notable strength of this study is the high quality and duration of echocardiographic follow-up, with serial imaging data available at 1, 3, 12, 24, 36, and up to 58 months postoperatively. This level of longitudinal assessment is uncommon in retrospective valve studies conducted at single centers, providing insight into the mid-term performance and durability of sutureless valves in real-world surgical practice. The consistency of imaging protocols and follow-up intervals enhances internal validity, providing robust data to complement findings from larger, multicenter trials. Future research could build upon these findings by incorporating matched cohort designs or contributing data to multicenter registries to improve statistical rigor and external generalizability.

## 5. Conclusions

This single-center retrospective comparative cohort study confirms that both conventional and minimally invasive AVR with the Perceval sutureless valve are safe and efficient, with satisfactory mid-term outcomes. Operative times and survival trends were favorable in the Perceval group, particularly beyond six months. The results also reflect the influence of the institutional learning curve, underscoring the importance of surgical experience in optimizing outcomes. Although the study’s size and design limit generalizability, the extended echocardiographic follow-up provides valuable insight into valve performance. Future research should aim to enhance comparability through matched cohorts or multicenter collaboration to strengthen clinical relevance and broaden applicability.

## Figures and Tables

**Table 1:** Preoperative patient characteristics.

Characteristic	Sutureless (N = 74)	Stented (N = 159)	*p*-Value
Age (years)	72.61 ± 7.21	72.67 ± 7.19	NS
Female, n (%)	34 (45.9%)	69 (43.4%)	NS
Male, n (%)	40 (54.1%)	90 (56.6%)	NS
Weight (kg)	77.92 ± 8.40	75.82 ± 7.60	NS
Height (cm)	165.60 ± 14.50	166.86 ± 15.22	NS
BMI (kg/m^2^)	26.6 ± 4.67	28.3 ± 11.53	NS
BSA (m^2^)	1.81 ± 0.14	1.92 ± 0.19	NS
Procedure: AVR (isolated)	61 (82%)	127 (80%)	NS
Procedure: AVR + CABG	13 (18%)	32 (20%)	NS
Peak transvalvular gradient (mmHg)	76 ± 26	74 ± 23	NS
Mean transvalvular gradient (mmHg)	52 ± 17	52 ± 17	NS
Aortic valve area (cm^2^)	0.61 ± 0.15	0.90 ± 0.24	<0.05
Bicuspid aortic valve, n (%)	2 (2.7%)	8 (5.0%)	NS
NYHA class II	47 (63.5%)	89 (56.0%)	NS
NYHA class III	27 (36.5%)	70 (44.0%)	NS
Coronary artery disease, n (%)	16 (21.6%)	33 (20.7%)	NS
Hypertension, n (%)	70 (94%)	122 (76%)	<0.05
Diabetes mellitus, n (%)	33 (44%)	64 (40%)	NS
Chronic lung disease, n (%)	14 (19%)	32 (20%)	NS
Neurological disease, n (%)	9 (12%)	20 (12.5%)	NS
Renal impairment, n (%)	14 (19%)	22 (14%)	NS
Peripheral vascular disease, n (%)	11 (15%)	16 (10%)	NS
Dyslipidemia, n (%)	49 (66%)	63 (40%)	<0.05
Current/previous smoking, n (%)	50 (67.6%)	102 (64.1%)	NS
LVEF < 30%, n (%)	3 (4%)	5 (3%)	NS
LVEF 30–50%, n (%)	16 (22%)	28 (18%)	NS
LVEF > 50%, n (%)	55 (74%)	126 (79%)	NS
EuroSCORE-II	1.95 ± 0.84	1.76 ± 0.94	NS
STS score	1.80 ± 0.74	1.54 ± 0.64	NS

Legend: NS—non significant; BMI—body mass index; BSA—body surface area; NYHA—New York Heart Association functional class; LVEF—left ventricular ejection fraction; EuroSCORE-II—European System for Cardiac Operative Risk Evaluation II; STS—Society of Thoracic Surgeons risk score.

**Table 2:** Intraoperative parameters and early hospital (30-day) outcome.

Characteristic	Sutureless (N = 74)	Stented (N = 159)	*p*-Value
**CPB time (min)**			
AVR (isolated)	83.8 ± 20.6 (n = 61)	82.7 ± 21.8 (n = 127)	NS
AVR + CABG	120.3 ± 38.2 (n = 13)	101.4 ± 36.5 (n = 32)	< 0.05
Full sternotomy	96.4 ± 44.5 (n = 51)	97.6 ± 42.8 (n = 137)	NS
Upper mini-sternotomy	88.4 ± 21.4 (n = 17)	89.2 ± 43.6 (n = 22)	NS
Right anterior thoracotomy	94.0 ± 9.2 (n = 6)	— (n = 0)	—
**ACC time (min)**			
AVR (isolated)	54.5 ± 14.6 (n = 61)	56.8 ± 11.6 (n = 127)	NS
AVR + CABG	92.1 ± 29.3 (n = 13)	104.5 ± 29.6 (n = 32)	< 0.05
Full sternotomy	65.8 ± 27.6 (n = 51)	67.6 ± 22.8 (n = 137)	NS
Upper mini-sternotomy	53.6 ± 15.8 (n = 17)	55.4 ± 13.7 (n = 22)	NS
Right anterior thoracotomy	64.7 ± 5.9 (n = 6)	— (n = 0)	—
**Distal anastomoses (CABG)**			
1 graft	5 (6.8%)	11 (6.9%)	NS
2 grafts	3 (4.0%)	6 (3.8%)	NS
3 grafts	6 (8.1%)	15 (9.4%)	NS
**Complications**			
Paravalvular leak (significant)	3 (4.0%)	2 (1.3%)	NS
Neurological dysfunction	2 (2.7%)	2 (1.3%)	NS
Thrombocytopenia	9 (12.2%)	11 (6.9%)	NS
Re-exploration for bleeding	4 (5.4%)	11 (6.9%)	NS
Permanent pacemaker required	4 (5.4%)	5 (3.1%)	NS
24 h chest tube output (mL)	405 ± 93	494 ± 102	NS
**ICU stay (days)**	2.4 ± 1.9	2.7 ± 2.1	NS
**Hospital stays (days)**	7.6 ± 3.6	8.1 ± 2.3	NS
**30-day (hospital) mortality**	4 (5.9%)	10 (6.3%)	NS

Legend: AVR—aortic valve replacement; CABG—coronary artery bypass grafting; ICU—intensive care unit.

**Table 3:** Late postoperative and mid-term (36-month) follow-up outcomes.

Characteristic	Sutureless (N = 74)	Stented (N = 159)	*p*-Value
Stroke, n (%)	0 (0%)	0 (0%)	NS
Endocarditis, n (%)	0 (0%)	3 (1.9%)	NS
Neurological event, n (%) *	3 (4.0%)	6 (3.8%)	NS
Thrombocytopenia, n (%)	15 (20.3%)	17 (10.7%)	<0.05
Paravalvular leak (trivial), n (%)	4 (5.4%)	5 (3.1%)	NS
Peak transvalvular gradient (postop, mmHg)	22.5 ± 8.1	24.5 ± 8.7	NS
Mean transvalvular gradient (postop, mmHg)	11.2 ± 4.3	12.6 ± 5.3	NS
Peak transvalvular gradient (follow-up, mmHg)	19 ± 2	20 ± 2.1	NS
Mean transvalvular gradient (follow-up, mmHg)	9 ± 2	10 ± 2.2	NS
NYHA class I (latest)	48 (64.8%)	92 (57.8%)	NS
NYHA class II (latest)	26 (35.2%)	67 (42.2%)	NS

Legend: * includes transient ischemic attack or another neurologic dysfunction.

**Table 4:** Postoperative survival during follow-up.

Months After AVR	Overall Survival (%)	Stented Group (%)	Sutureless Group (%)
1	94.0	93.7	94.6
3	93.5	93.1	94.6
12	90.1	88.4	91.6
24	85.0	83.7	88.3
36	80.2	76.8	88.3
48	—	—	88.3
58	—	—	88.3

Legend: “—” indicates no patients at risk (no data) in the stented valve group after 36 months.

## Data Availability

Raw data supporting the conclusions of this study will be made available by the authors upon request.

## Supplementary Materials

The following supporting information can be downloaded at https://www.mdpi.com/article/10.3390/jcdd12080308/s1: Table S1: Implanted Valve Models and projected effective orifice areas (EOAs) provided by the manufacturers derived from echocardiographic (in vivo) measurements.

## Abbreviations

The following abbreviations are used in this manuscript:

ACC	Aortic Cross-Clamp
AS	Aortic Stenosis
AVR	Aortic Valve Replacement
AVR + CABG	Aortic Valve Replacement plus Coronary Artery Bypass Grafting
BMI	Body Mass Index
BSA	Body Surface Area
CABG	Coronary Artery Bypass Grafting
CPB	Cardiopulmonary Bypass
CT or MDCT	(Multi-Detector) Computed Tomography
DVI	Doppler Velocity Index
EF/LVEF	Ejection Fraction/Left Ventricular Ejection Fraction
ICU	Intensive Care Unit
LVEF	Left Ventricular Ejection Fraction
NYHA	New York Heart Association
SD	Standard Deviation
STS	Society of Thoracic Surgeons
SU-AVR	Sutureless Aortic Valve Replacement
TEE	Transesophageal Echocardiography
TTE	Transthoracic Echocardiography
Vmax	Peak Aortic Jet Velocity
