# Supplementary material for: Comparison of Surgical Treatment Outcomes in Patients with Symptomatic Severe Aortic Valve Stenosis Using the Perceval Sutureless Bioprosthesis Versus a Conventional Biological Valve
Source: J Cardiovasc Dev Dis. 2025 Aug 13;12(8):308. doi: 10.3390/jcdd12080308 (PMC12387097; doi:10.3390/jcdd12080308)
Supplement: Supplementary file 1 [file jcdd-12-00308-s001.zip › jcdd-3772719-supplementary.pdf]

Table S1 - Implanted Valve Models and Effective Orifice Areas (EOAs)

Valve Model	Manufacturer	Tissue Type	Valve Size	EOA (cm²)	No(%)
**Sutureless**					
Perceval S (Small)	Corcym	Bovine pericardial	19–21 mm	1.0	15 (20.3)
Perceval M (Medium)	Corcym	Bovine pericardial	21–23 mm	1.2	20 (27.0)
Perceval L (Large)	Corcym	Bovine pericardial	23–25 mm	1.4	30 (40.5)
Perceval XL (X-Large)	Corcym	Bovine pericardial	25–27 mm	1.6	9 (12.2)
**Total**					**74 (100%)**
**Stented**					
Trifecta	St.Jude/Abbott	Bovine pericardial	19 mm	1.4	3 (1.9)
Trifecta	St.Jude/Abbott	Bovine pericardial	21 mm	1.6	63 (39.6)
Trifecta	St.Jude/Abbott	Bovine pericardial	23 mm	1.8	35 (22.0)
Trifecta	St.Jude/Abbott	Bovine pericardial	25 mm	2.1	9 (5.8)
Trifecta	St.Jude/Abbott	Bovine pericardial	27 mm	2.3	-
Epic Max	Abbott	Porcine	19 mm	1.2	1 (0.6)
Epic Max	Abbott	Porcine	21 mm	1.4	24 (15.2)
Epic Max	Abbott	Porcine	23 mm	1.6	10 (6.4)
Epic Max	Abbott	Porcine	25 mm	1.8	2 (1.2)
Epic Max	Abbott	Porcine	27 mm	2.0	-
Crown PRT	Sorin/LivaNova	Porcine	19 mm	1.1	2 (1.2)
Crown PRT	Sorin/LivaNova	Porcine	21 mm	1.3	4 (2.5)
Crown PRT	Sorin/LivaNova	Porcine	23 mm	1.5	2 (1.2)
Crown PRT	Sorin/LivaNova	Porcine	25 mm	1.7	2 (1.2)
Crown PRT	Sorin/LivaNova	Porcine	27 mm	1.9	-
Hancock II	Medtronic	Porcine	19 mm	1.0	-
Hancock II	Medtronic	Porcine	21 mm	1.2	1 (0.6)
Hancock II	Medtronic	Porcine	23 mm	1.4	1 (0.6)
Hancock II	Medtronic	Porcine	25 mm	1.6	-
**Total**					**159 (100%)**
